# Genetic Analysis of Lice Supports Direct Contact between Modern and Archaic Humans

**DOI:** 10.1371/journal.pbio.0020340

**Published:** 2004-10-05

**Authors:** David L Reed, Vincent S Smith, Shaless L Hammond, Alan R Rogers, Dale H Clayton

**Affiliations:** **1**Florida Museum of Natural History, Dickinson Hall, University of FloridaGainesville, FloridaUnited States of America; **2**Graham Kerr Building, DEEB, IBLS, University of GlasgowGlasgowScotland; **3**Department of Biology, University of UtahSalt Lake City, UtahUnited States of America; **4**Department of Anthropology, University of UtahSalt Lake City, UtahUnited States of America

## Abstract

Parasites can be used as unique markers to investigate host evolutionary history, independent of host data. Here we show that modern human head lice, *Pediculus humanus,* are composed of two ancient lineages, whose origin predates modern Homo sapiens by an order of magnitude (ca. 1.18 million years). One of the two louse lineages has a worldwide distribution and appears to have undergone a population bottleneck ca. 100,000 years ago along with its modern H. sapiens host. Phylogenetic and population genetic data suggest that the other lineage, found only in the New World, has remained isolated from the worldwide lineage for the last 1.18 million years. The ancient divergence between these two lice is contemporaneous with splits among early species of *Homo,* and cospeciation analyses suggest that the two louse lineages codiverged with a now extinct species of *Homo* and the lineage leading to modern *H. sapiens.* If these lice indeed codiverged with their hosts ca. 1.18 million years ago, then a recent host switch from an archaic species of *Homo* to modern H. sapiens is required to explain the occurrence of both lineages on modern *H. sapiens.* Such a host switch would require direct physical contact between modern and archaic forms of *Homo.*

## Abstract 2

A phylogenetic analysis reveals that humans have two types of head lice and that one must have switched from an ancient to a modern human host, suggesting these humans had contact

## Introduction

One of the most intensely debated topics in evolutionary biology pertains to the origin of modern *Homo sapiens.* The debate concerns the precise manner in which anatomically modern humans arose from archaic ancestors. Empirical studies tend to support one of two prominent models of human origins, the Recent African Replacement model (Stringer and Andrews 1988) or the Multiregional Evolution model (Wolpoff et al. 1994). The Recent African Replacement model, as originally proposed, suggests that modern humans arose from an archaic ancestor in Africa ca. 130,000 years ago, and then replaced archaic humans in Asia, Africa, and Europe without introgression between archaic and modern humans. The Multiregional Evolution model (as proposed by Wolpoff et al. [1994] and revisited by Wolpoff et al. [2000]) suggests that gene flow existed not only among populations of modern *Homo sapiens,* but also between modern H. sapiens and archaic forms of *Homo* (e.g., Homo neanderthalensis and Homo erectus), which led to some degree of regional continuity. Both models can be subdivided into many variants. There are two common variants of the Multiregional Evolution model. In one variant, the transition from archaic to modern humans occurs incrementally across a large geographic region (i.e., both within and outside Africa); in the other variant, the transition from archaic to modern humans arises first in Africa then spreads through gene flow outside of Africa. This latter variant is very similar to a Diffusion Wave model recently put forth by Eswaran (2002). Both types of models of human origins (the Recent African Replacement and Multiregional Evolution models) have been examined with both human fossil and genetic data, but no single model or variant has been supported by all the data.

Fossils provide the only source of data available for most species of archaic humans and are therefore crucial to understanding the origin of modern humans. Unfortunately, missing taxa and fragmentary fossils limit our ability to reconstruct human evolutionary history based solely on fossil data. Molecular (DNA sequence) data have provided additional insight into the recent evolutionary history of humans, but these data are limited mainly to extant human populations. Ancient DNA was recently sequenced from H. neanderthalensis (Krings et al. 1997, 1999, 2000) and a 24,000-year-old specimen of modern H. sapiens (Caramelli et al. 2003), but even these ancient DNA studies do not agree on hypotheses of modern human origins (Templeton 2002; Serre et al. 2004). Only a few ancient specimens have been examined molecularly, and additional sequences are slow to emerge. Furthermore, DNA may never be retrieved from some specimens because it is difficult, if not impossible, to liberate sequenceable DNA from poorly preserved (Krings et al. 1997) or very old (Paabo and Wilson 1991) fossil material. Therefore, the degree to which we can reconstruct human evolutionary history depends, in part, upon additional types of data.

Several recent studies have inferred portions of human evolutionary history from the evolutionary history of their parasites (Chan et al. 1992; Ho et al. 1993; Ong et al. 1993; Escalante et al. 1998; Ashford 2000; Leal and Zanotto 2000; Hoberg et al. 2001). Parasites can be a powerful tool for reconstructing host evolutionary history because they provide data that are independent of host data. For example, human papillomaviruses (Chan et al. 1992; Ho et al. 1993; Ong et al. 1993), tapeworms (Hoberg et al. 2001), and malarial parasites (Escalante et al. 1998) each have evolutionary origins in Africa, consistent with most human fossil and molecular data. Human T-cell leukaemia/lymphoma virus (HTLV) sequences show that most human viral strains are closely related to those of Old World apes and monkeys (Leal and Zanotto 2000). In contrast, some Native American strains of HTLV have closer affinities to viral strains from Asian primates, suggesting a dual origin for this virus in humans (Leal and Zanotto 2000 and references therein). Ashford (2000) recently reviewed the use of parasites as markers of human evolutionary history, pointing out that five parasites of humans (lice, tapeworms, follicle mites, a protozoan, and bedbugs) have closely related taxonomic pairs that suggest periods of host geographic isolation. Unfortunately, none of these five pairs has been studied rigorously with the primary goal of inferring host evolutionary history. Of these parasites, the ones most likely to provide the greatest insight into human evolutionary history are those that are known to have had a long-term coevolutionary association with their hosts, such as lice *(Insecta: Phthiraptera)* (Page 2003).

Lice are obligate parasites of mammals or birds that complete their entire life cycle on the body of the host; they cannot survive more than a few hours or days off the host (Buxton 1946). Mammal lice are closely tied to their hosts in both ecological (Reed and Hafner 1997) and evolutionary (Hafner et al. 1994) time. The lice found on primates are quite host specific, with most species occurring only on a single species of host (Durden and Musser 1994). Host specificity is reinforced by the fact that primate lice require direct physical contact between hosts for transmission (Buxton 1946; Durden 2001; Canyon et al. 2002; Burgess 2004). Host specificity often goes hand in hand with long-term coevolutionary patterns between hosts and parasites (Page 2003), making primate lice excellent candidates for inferring host evolutionary history. Humans are parasitized by two species of lice: head/body lice *(Pediculus humanus),* the focus of this paper, and pubic lice*(Pthirus pubis),* which serve as a phylogenetic outgroup in this study. P. humanus is found in two forms (head and body lice) that are morphologically similar, but ecologically distinct. Body lice live primarily in clothing and move onto the skin to feed once or twice a day. Head lice are confined to the scalp and feed more frequently. Body lice vector the bacteria responsible for epidemic typhus, trench fever, and relapsing fever; head lice are not known to vector any agent of human disease under natural conditions (Buxton 1946).

Recent molecular work by Leo et al. (2002) showed that, despite the ecological differences between head and body lice, the two forms are not genetically distinct. Kittler et al. (2003) confirmed this finding but also discovered two deeply divergent clades within P. humanus that are uncorrelated with the head and body louse forms. The divergent clades of lice stand in contrast to mitochondrial sequence data from extant human populations, which coalesce to a single lineage very rapidly. The shallow coalescence in human mitochondrial sequence data is likely the result of a recent population bottleneck and subsequent population expansion (Rogers and Harpending 1992), which obscures much of the evolutionary history of humans prior to the bottleneck. The deep divergences within P. humanus have the potential to reveal aspects of human evolutionary history that cannot be recovered from human DNA markers.

We reconstructed the evolutionary history of P. humanus and several outgroup taxa using both morphological and molecular data. First, we used louse morphological data to test for patterns of cospeciation between primate lice and their hosts. Then we collected molecular data from a subset of the same taxa and calculated divergence dates for nodes in the louse phylogeny. This broad phylogenetic approach allowed us to date the origin of the human louse, *P. humanus,* and to date the two divergent lineages within the species. Finally, we collected population genetic data for P. humanus to compare with population-level characteristics of extant humans. Taken together, our phylogenetic and population-level data provide a well-resolved picture of the evolutionary history of *P. humanus,* which can be used to indirectly infer human evolutionary history. Specifically, we compared three distinct models of modern human origins (Recent African Replacement without Introgression, Multiregional Evolution, and Diffusion Wave) to see which model best fits the data from human lice.

## Results

### Phylogenetic Analyses and Divergence Estimates

**Figure 1:**
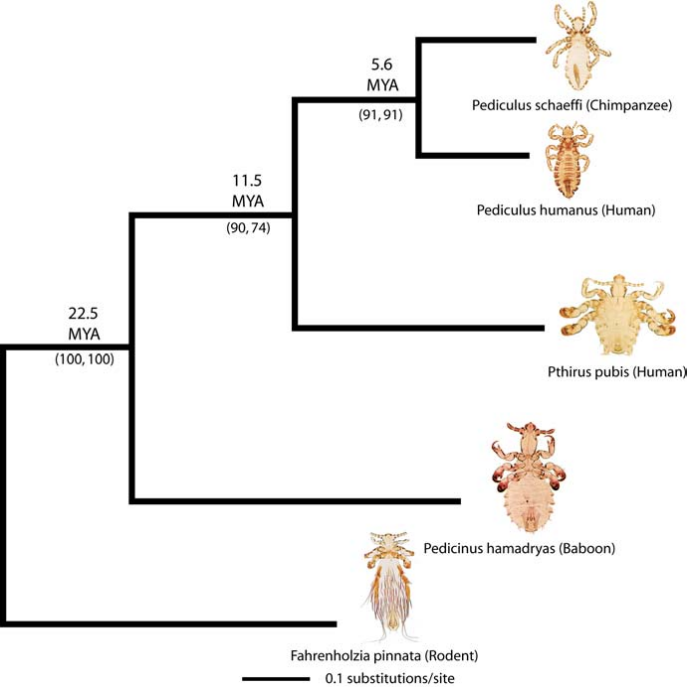
Phylogeny of Primate Lice from Morphological and Molecular Data The phylogeny is a strict consensus of morphology and a 1,525-bp fragment of COI and Cytb. Branch lengths were determined from the molecular data. Numbers in parentheses are bootstrap values from molecular and morphological data, respectively. Divergence dates are direct estimates from mtDNA data (see text). Louse images from light microscopy were taken by VSS.

Both the morphological and molecular data sets produced a single phylogenetic relationship for the louse species in Figure 1. The phylogeny shows that *Pediculus* species on chimpanzees and humans are sister taxa, which together with *Pthirus* form a clade that is sister to *Pedicinus,* the most basal member of the ingroup (Figure 1). Bootstrap support for these relationships is high. Reconciliation analysis using Treemap v. 2.0 (M. A. Charleston and R. D. M. Page, software distributed by authors) revealed significant congruence (*p* < 0.01) between the louse and primate phylogenies, thus validating the assumption of cospeciation (Kittler et al. 2003). Reconciliation analysis using Treemap showed four cospeciation events and one host switch. One particular node of cospeciation determined that as cercopithecoid and hominoid primates diverged 20–25 million years ago (MYA) (Benefit 1993; Leakey et al. 1995), *Pedicinus* diverged from the lineage leading to *Pediculus* and *Pthirus.* Since the nodes of cospeciation in congruent host and parasite trees are contemporaneous, the louse tree can be calibrated using the host tree.

We used the date of 22.5 ± 2.5 MYA to calibrate the split between *Pedicinus* and *Pthirus* + *Pediculus* in the louse tree. This, in turn, yielded a divergence time of 11.5 MYA for the *Pthirus*/*Pediculus* split and 5.6 MYA for the split between Pediculus schaeffi and P. humanus (Table 1). Our estimated divergence between chimp and human lice (5.6 MYA) is strikingly similar to the 5.5 MYA estimates for the chimp/human divergence based on both mitochondrial and nuclear sequence data (Stauffer et al. 2001). To test the original calibration date of 22.5 MYA, we used the molecular estimate of the chimp/human split (5.5 MYA; Stauffer et al. 2001) to reverse calibrate the louse tree. This younger calibration point resulted in divergence estimates that were nearly identical to those from the previous calibration. For example, the 5.5 MYA calibration resulted in an estimated divergence of 22.65 MYA for the split between *Pedicinus* and *Pthirus* + *Pediculus.* Estimates of divergence time error were calculated from bootstrapped data sets (Table 1). Other studies have shown that louse mitochondrial DNA (mtDNA) sequences evolve at a rate two to three times faster than that of host sequence rates (Page 1996; Page et al. 1998). The lice in this study are evolving at ca. 2.3 times the rate of their primate hosts, when nucleotide substitutions are estimated under a best-fit model of sequence evolution.

**Table 1:**
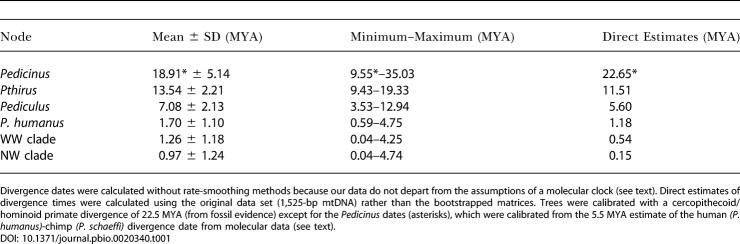
Mean (± Standard Deviation), Minimum, and Maximum Estimates of Divergence Times (in Millions of Years) of Louse Lineages from 100 Bootstrapped Data Matrices

Divergence dates were calculated without rate-smoothing methods because our data do not depart from the assumptions of a molecular clock (see text). Direct estimates of divergence times were calculated using the original data set (1,525-bp mtDNA) rather than the bootstrapped matrices. Trees were calibrated with a cercopithecoid/hominoid primate divergence of 22.5 MYA (from fossil evidence) except for the *Pedicinus* dates (asterisks), which were calibrated from the 5.5 MYA estimate of the human *(P. humanus)*-chimp *(P. schaeffi)* divergence date from molecular data (see text)

**Figure 2:**
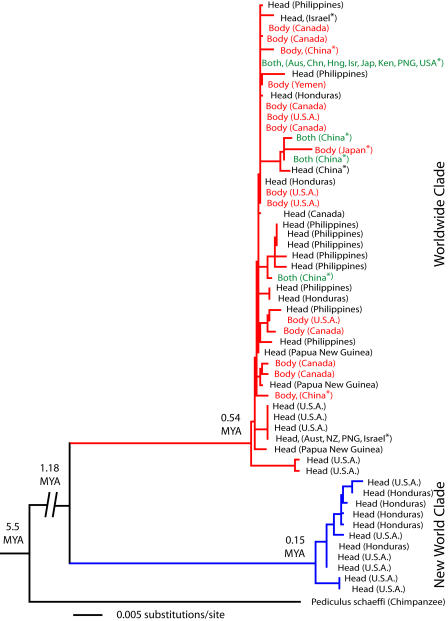
Molecular Phylogeny of P. humanus from Geographically Diverse Human Populations This species exhibits distinct “head” and “body” forms, which differ in ecology, and slightly in size. Head lice (black lettering) are smaller than body lice (red lettering) and are confined to the scalp, whereas body lice live primarily in clothing. Haplotypes shown in green were found in both head and body lice. There are no fixed genetic differences between the head and body forms, suggesting a lack of reproductive isolation, despite the fact that the two forms can be distinguished using discriminant function analysis of morphological data. These results are consistent with experimental data showing that head lice can transform morphologically into body lice within a few generations (Levene and Dobzhansky 1959). The Worldwide clade (red branches) shares a MRCA ca. 0.54 MYA and the geographically restricted New World clade (blue branches) has a much younger MRCA, ca. 0.15 MYA. Asterisks denote samples from Leo et al. (2002)

**Figure 3:**
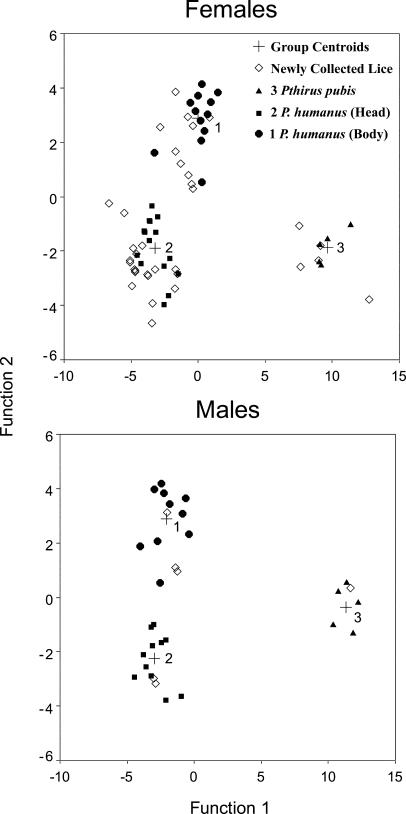
Plot of the First and Second Canonical Discriminant Functions for Specimens of Adult Head/Body Lice *(P. humanus)* and Pubic Lice *(Pthirus pubis)* Solid points denote reference specimens of known identity acquired from museum collections. Unfilled points denote newly collected specimens used in the molecular analyses. In all cases the discriminant function analysis successfully classified each unknown case with a probability of >0.95.

Phylogenetic analysis revealed two divergent clades within P. humanus (6% uncorrected sequence divergence for cytochrome oxidase subunit I [COI] and cytochrome *b* [Cytb]). One of the two lineages in our data set is worldwide (WW) in distribution (Figure 2, Worldwide clade), contains both head and body louse forms, as determined by discriminant function analysis (Figure 3), and has a most recent common ancestor (MRCA) 0.54 MYA (Table 1). Even within this WW clade head and body lice are not reciprocally monophyletic, and a constraint to enforce such monophyly can be rejected using a Shimodaira-Hasegawa test (*p* < 0.01) (Shimodaira and Hasegawa 1999). The other lineage (Figure 2, New World clade) is restricted to the New World (NW), contains only the head louse form, and has a MRCA only 0.15 MYA. The MRCA of all P. humanus was 1.18 MYA, which predates by a considerable margin the origin of modern H. sapiens based on mtDNA (≤0.20 MYA; Cann et al. 1987; Vigilant et al. 1991; Ingman et al. 2000) as well as fossil evidence (0.15–0.16 MYA; White et al. 2003).

**Figure 4:**
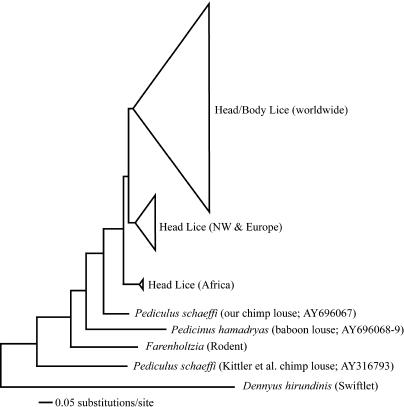
Neighbor-Joining Tree Using a Best-Fit Model of Nucleotide Substitution (Tamura-Nei + Γ) for a Combined Data Set of Cytb Sequences from Our Study and from Kittler et al. (2003) The clades of P. humanus identified by Kittler et al. (2003) are nearly identical to those from our data, with the exception of their basal African clade, which was not represented in our data set. One clade contains both head lice and body lice and is WW in distribution. Another clade is comprised solely of head lice from the NW (our data) and Europe (samples from Kittler et al. 2003), and the most basal clade contains isolates 4, 18, and 33 from Kittler et al. (2003), which are head lice from Africa. The size of the triangles representing the three clades are proportional in size to the number of taxa within the clade. This phylogeny is rooted with a divergent louse, *Dennyus hirundinus,* which is a bird louse in the suborder *Amblycera.* Note the placement of the Kittler et al. (2003) specimen of *P. schaeffi,* which falls outside all other primate lice and the rodent louse *Fahrenholzia.*

Our estimate of the age of the MRCA for P. humanus (1.18 MYA) is much older than that reported by Kittler et al. (2003), which was only 0.53 MYA based on mtDNA. Their estimate of 0.53 MYA was determined using a mtDNA sequence from a specimen of the chimp louse, *P. schaeffi,* that is quite aberrant when compared to other primate lice. Phylogenetic analysis of the Kittler et al. Cytb data (downloaded from GenBank), combined with our own data, shows that the Kittler et al. sequence for P. schaeffi is 40% divergent from P. humanus and 40% divergent from our own sequence of *P. schaeffi.* Phylogenetic analysis places their specimen of P. schaeffi outside all other primate lice and even outside the rodent louse (Figure 4), whereas our specimen of P. schaeffi is sister to *P. humanus,* based on both morphology and molecular data. We think that the Kittler et al. specimen has been attributed to the species P. schaeffi in error. In contrast to the mitochondrial data reported by Kittler et al. (2003), our analysis of their nuclear elongation factor (EF1-alpha) sequences produces a MRCA for P. humanus that is ca. 2 MYA. Similarly, 18S rRNA sequences for P. humanus from Yong et al. (2003), combined with an 18S rRNA sequence from *P. schaeffi,* provide a MRCA for P. humanus that is ca. 2 MYA (for GenBank accession numbers, see Supporting Information). Together, these mitochondrial and nuclear markers support a MRCA for P. humanus greater than 1.18 MYA, which is an order of magnitude older than the MRCA for its human host.

### Population Genetic Analysis of P. humanus

We can calculate an expected date of mitochondrial coalescence for P. humanus if we assume for the moment that the entire population of lice mated at random (i.e., panmixia). The estimate of expected coalescence is based on the effective female population size (N_ef_), which was estimated from the sample of all P. humanus specimens to be 1.1 million female lice from the equation Θ = 2N_ef_μ. The estimate of N_ef_ provides an expected coalescence time for the two divergent mitochondrial lineages of P. humanus of 1.10 million generations or ca. 0.11 MYA, which is an order of magnitude younger than the observed divergence time of 1.18 MYA. In a large randomly mating population consisting of 1.1 million female lice, one would expect to maintain two distinct haplotypes for only ca. 0.11 million years (MY). This suggests that we can reject panmixia if we assume that N_ef_ prior to the bottleneck was roughly similar to what we see today. If estimates of N_ef_ were drastically higher (ca. 60 million female lice) prior to the bottleneck, then expected time to coalescence could be much longer. The F_st_ value, a measure of genetic population differentiation, calculated for the WW and NW clades was 0.96, indicating substantial population structure, which also supports the rejection of panmixia.

The WW clade of P. humanus shows evidence of a recent population expansion (Fu and Li's D* = −2.80 [Fu and Li 1993]; *p* < 0.02). We estimated the date of this population expansion from the mismatch distribution. The estimate was calculated by comparing the average pairwise difference within the WW clade of P. humanus (4.21 mutations) to the pairwise difference between P. humanus and P. schaeffi (220 mutations), which diverged 5.6 MYA. The population expansion of the WW clade is estimated to be 0.11 MYA, similar to the estimated date of population expansion of modern humans out of Africa ca. 0.10 MYA (di Rienzo and Wilson 1991; Rogers and Harpending 1992; Harpending et al. 1993). In contrast, the NW clade of P. humanus does not exhibit the signature of a recent population expansion (Fu and Li's D* = 0.17), but instead shows a more stable population size.

### Contemporaneous Divergences in *Pediculus* and Archaic *Homo* spp.

The age of the MRCA of P. humanus dates to 1.18 MYA (for mtDNA), which is roughly midway between the estimated ages of H. neanderthalensis (0.60 MY) and H. erectus (1.8 MY). We used a maximum likelihood (ML) analysis to test whether our two divergent lineages of lice could have diverged in tandem with H. sapiens and H. neanderthalensis (Neandertals). H. neanderthalensis is the only other species of *Homo* for which DNA sequence data are available (Krings et al. 1999). The test evaluated whether relative branch lengths (scaled according to mutation rate) in the host tree, specifically for the branch between H. sapiens and *H. neanderthalensis,* are consistent with the parasite DNA sequence data (Huelsenbeck et al. 1997). In cospeciating assemblages, host and parasite branch lengths are highly correlated due to a shared evolutionary history (Page 1996). A likelihood ratio test (LRT) rejected (*p* < 0.0001) the H. sapiens/H. neanderthalensis split as a node of cospeciation with the two clades of P. humanus because the branch length between H. sapiens and H. neanderthalensis is far too short to explain the louse DNA sequence data. In other words, the split between H. sapiens and H. neanderthalensis is too recent to have been contemporaneous with the divergence of the two lineages of lice. If one artificially lengthens the branch between H. sapiens and H. neanderthalensis to approximate the split between H. sapiens and H. erectus (anywhere from 1.2 to 1.8 MYA), the LRT fails to reject this hypothesis of cospeciation.

## Discussion

Morphological and molecular data agree that primates and their lice have been cospeciating for over 20 MY. Indeed, it is this cospeciation that permits us to use host fossil evidence to calibrate portions of the louse phylogenetic tree. This has resulted in the discovery of two extant lineages of human lice that diverged 1.18 MYA. This ancient divergence is surprising because humans, and presumably their lice, are thought to have passed through a population bottleneck ca. 0.05–0.10 MYA (Rogers and Harpending 1992). Such bottlenecks reduce genetic diversity by eliminating uncommon haplotypes, thereby making it less likely that multiple haplotypes survive bottleneck events. For example, mtDNA sequences from human populations coalesce to a single lineage very quickly (≤0.20 MYA), presumably the result of the population bottleneck. The deep divergences found in P. humanus could conceivably be the result of sequencing a nuclear copy of a mitochondrial gene. However, several lines of evidence strongly suggest otherwise. Because we amplified two different mitochondrial genes (COI and Cytb) that show the same divergent lineages and similar percent sequence divergences, copies of both mitochondrial genes would have had to enter the nucleus simultaneously, which is unlikely. In addition, we amplified each gene with a nested set of overlapping primers, and we never amplified more than one gene copy, even during bouts of cloning. Nucleotide base composition for our COI and Cytb data do not deviate from the mean values for all louse COI and Cytb sequences in GenBank (unpublished data), which would not be the case for a nuclear copy of a mitochondrial gene. Finally, the deep divergences seen in our mitochondrial genes are confirmed by preliminary analyses of nuclear data (EF1-alpha and 18S rRNA, unpublished data). Therefore, we are confident that the DNA sequences used in this study are mitochondrial in origin, and we must attempt to explain the occurrence of such ancient mitochondrial haplotypes in human lice.

### Gene Trees and Ancient Polymorphisms

Gene trees (e.g., mitochondrial lineages) can be considerably older than species trees, and therefore our louse mitochondrial lineages could predate the actual origin of the species P. humanus (i.e., its speciation time). It is useful to determine an expected time to coalescence from the estimated N_ef_ of 1.1 million female lice, even though this estimate seems high for a parasite of humans, who themselves have had very small effective population sizes (as few as 10,000 individuals) and recently went through a population bottleneck (Rogers and Harpending 1992). Although we do not necessarily expect human and louse effective population sizes to be directly correlated, it is difficult to imagine that humans could have maintained such a large effective population of lice during a bottleneck event. Regardless, the expected time to coalescence was estimated to be 0.10 MYA, an order of magnitude younger than the observed divergence time of 1.18 MYA. The deeper gene tree that our data provide also could have been produced either by balancing selection or by subdivision of the louse population into several distinct groups with very limited gene flow. A Fu and Li test does not detect balancing selection when both lineages of P. humanus are evaluated together (*p* = 0.11); therefore, we must consider the alternative explanation of extensive population subdivision.

### Population Substructure and Host Geographic Isolation

Substantial isolation among populations of lice on modern H. sapiens could disrupt gene flow and allow the retention of very old lineages, making the age of P. humanus seem much older than it actually is. However, there is no evidence of such pervasive geographic isolation in the modern human hosts of these lice. Other species of lice have been shown to have substantial geographic substructure (i.e., isolation) even when hosts show no geographic isolation (Johnson et al. 2002). If populations of P. humanus are more highly subdivided than those of their hosts, then we might expect P. humanus to have retained ancient mitochondrial polymorphisms, even through host bottleneck events. One prediction of this hypothesis would be that both clades of P. humanus (the WW and NW clades) would show signs of the recent population expansion of humans during the last ca. 0.10 MY. However, only the WW clade shows evidence of this event, which very closely matches the timing of human population expansion.

Because the WW clade is commonly found worldwide, and shows a population expansion concurrent with that of modern *H. sapiens,* we conclude that this lineage has a common evolutionary history with modern *H. sapiens.* In contrast, the NW clade appears to have diverged from the WW lineage 1.18 MYA, and has had a distinctly different evolutionary history. We are left unable to explain the retention of two ancient louse lineages, each with a different evolutionary history, within the confines of a single host, modern *H. sapiens.* Given the history of cospeciation between primate lice and their hosts, it is necessary to look beyond modern H. sapiens to determine whether the two divergent lineages of P. humanus are legacies of a more ancient divergence.

### Contemporaneous Divergences in *Pediculus* and Archaic *Homo* spp.

ML analyses rejected H. neanderthalensis as having diverged from H. sapiens contemporaneously with the two divergent lineages of lice. The mitochondrial MRCA of Neandertals and humans is 0.60 MYA (Krings et al. 1997), which is only about half as old as the MRCA of the two ancient lineages of *P. humanus,* 1.18 MYA. The same ML test failed to reject the codivergence of these lice with H. erectus and H. sapiens when their divergence was set anywhere between 1.2 and 1.8 MYA. Therefore, the deep divergence within P. humanus is entirely consistent with a cospeciation event within the genus *Homo* ca. 1.2–1.8 MYA, but not 0.60 MYA. Unfortunately, no DNA sequence data exist for H. erectus or any other archaic species of *Homo* to enable a more direct test of cospeciation.

**Figure 5:**
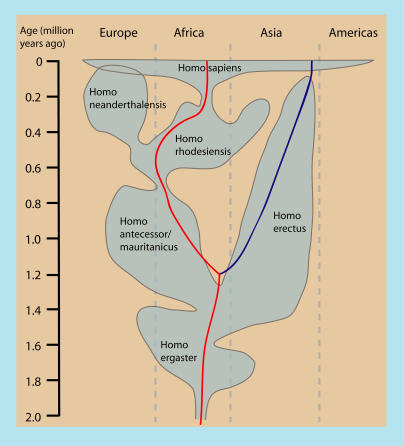
Temporal and Geographical Distribution of Hominid Populations Redrawn from Stringer (2003) This figure depicts one view of human evolutionary history based on fossil data. Other interpretations differ primarily in the taxonomy and geographical distribution of hominid species. The temporal distribution of the two divergent lineages of P. humanus is superimposed on the hominid tree to show host evolutionary events that were contemporaneous with the origin of *P. humanus.* Whereas the NW lineage is depicted on H. erectus in this figure, several alternative hypotheses are consistent with our data when other evolutionary histories of hominids are considered (unpublished data). The WW clade is shown in red and the NW clade in blue (see text for descriptions of clades).

There is much debate regarding the past 2 MY of hominid evolution. However, one area of broad agreement is that, prior to 2 MYA, our ancestors were confined to Africa, then left the continent ca. 1.8 MYA. This first migration out of Africa resulted in archaic species of *Homo* that were widespread in distribution, and at times both contemporaneous with, and geographically isolated from, the lineage leading to modern H. sapiens (Figure 5). The 1.18 MY of isolation required to preserve the two ancient louse lineages must have occurred, in part, among these archaic species of *Homo.* It should be noted here that some interpretations of the Multiregional Evolution model do not necessarily consider modern H. sapiens to be a distinctly different species from archaic humans (e.g., H. erectus and H. neanderthalensis). We refer to them as “species” mostly for convenience of writing. Whereas the WW lineage has population genetic characteristics that are similar to those of modern *H. sapiens,* the geographically restricted NW lineage does not. It likely evolved on a now extinct species of *Homo* only to switch to modern H. sapiens very recently. For example, Figure 5 depicts one possible scenario where the NW lineage evolved on H. erectus and switched to modern *H. sapiens.* Interestingly, Hoberg et al. (2001) reported that two species of tapeworms of humans diverged ca. 0.78–1.71 MYA, and one of the two species, *Taenia asiatica,* is entirely restricted to Asia. This is consistent with the depiction in Figure 5, if one assumes that T. asiatica evolved on *H. erectus.* Although divergence dates are not available, it is intriguing that some Native American strains of HTLV have closer affinities to Asian primate strains than to other human strains of HTLV, suggesting an independent Asian origin of this virus in humans. One must still explain how these parasites came to be on modern *H. sapiens,* but taken together, the parasitological evidence (especially the deep divergences in tapeworms and lice) suggests that they might have evolved on H. erectus and switched recently to *H. sapiens.* If true, this implies that H. erectus was contemporaneous with modern H. sapiens in eastern Asia, as suggested by Swisher et al. (1996), and it begs a discussion of recent human origins.

### Recent Human Origins

Explanations of modern human origins are dominated by two competing models, Recent African Replacement and Multiregional Evolution. The Recent African Replacement model assumes that anatomically modern H. sapiens arose as the result of a speciation event ca. 0.13 MYA, and then replaced without introgression (i.e., admixture) non-African archaic humans (e.g., H. neanderthalensis and H. erectus). In contrast, the sensu stricto Multiregional Evolution model assumes that modern H. sapiens evolved from early African descendants (up to 2 MYA). Characteristics of modernity were spread geographically through intercontinental gene flow, but local regional characteristics were maintained through admixture between modern and archaic forms (Wolpoff et al. 1994). Most debates on modern human origins in the recent literature focus on one central question: “Was there admixture (i.e., introgression) between modern and archaic humans?” Both the Recent African Replacement and Multiregional Evolution models have been proposed with numerous variations that include introgression, one of which was recently put forth by Eswaran (2002). Eswaran's Diffusion Wave model proposes that a diffusion wave of modern H. sapiens left Africa (ca. 0.13 MYA) and replaced archaic humans through a process of introgression, natural selection, and gradual demic expansion.

The evolutionary history of P. humanus is somewhat consistent with all three models of modern human origins mentioned above; however, the number of ad hoc assumptions required to reconcile host and parasite evolutionary histories varies among the three views of human origins. Each model can account for the deep divergence between the two clades of P. humanus because each recognizes divergences between archaic species of *Homo* ca. 2 MYA. However, the model that best fits the louse data must account not only for the 1 MY of isolation between archaic and modern forms of lice, but also for a recent population expansion in just one louse lineage, the WW clade. The model must also explain how archaic louse DNA might have been incorporated into the lice of modern *H. sapiens.*

The sensu stricto model of Multiregional Evolution (Wolpoff et al. 1994) predicts continual gene flow between the geographically separated populations of humans following their early migration out of Africa (ca. 2 MYA), which is inconsistent with the louse data. This intercontinental gene flow among humans is required in the Multiregional Evolution model to maintain the continuity of the species *H. sapiens.* This scenario does not provide the louse populations with the degree of isolation necessary (ca. 1.18 MY) to maintain the two divergent louse lineages, unless we assume that gene flow between human populations was considerably greater than gene flow among their populations of lice. There is no reason to assume such a disparity in gene flow between hosts and parasites. The Multiregional Evolution model also predicts that we should detect the same genetic fingerprint of recent population expansion in both clades of *P. humanus,* which we do not.

The Recent African Replacement model provides the isolation necessary between archaic and modern forms, because it assumes that modern H. sapiens left Africa ca. 0.10 MYA, more than a million years after archaic species of *Homo* left Africa, and that the modern and archaic humans remained distinct (i.e., no introgression). Furthermore, it explicitly assumes a recent population expansion in modern *H. sapiens,* which would account for the population expansion seen in our WW clade. However, in the strict sense, this model also predicts that modern H. sapiens replaced archaic forms of humans without introgression (i.e., hybridization), which leaves no obvious mechanism for archaic louse DNA to reach the lice of modern *H. sapiens.* This lack of host introgression implies, but does not require, a lack of direct physical contact between modern and archaic humans. It is conceivable that direct contact between modern and archaic humans was sufficient to allow the lice to switch hosts without making the assertion that the hosts were interbreeding. Therefore, the Recent African Replacement model is fairly consistent with the louse data, so long as one assumes some level of direct contact (e.g., fighting, sharing/stealing of clothing, etc.) between modern and archaic humans.

Eswaran's Diffusion Wave model (2002) is similar to the Multiregional Evolution model in that it permits some level of introgression between modern and archaic humans. However, it is also similar to the Recent African Replacement model in that it assumes the same recent population expansion of modern humans out of Africa (ca. 0.10 MYA), thus providing both the isolation and population expansion necessary to accommodate our louse data. The additional assumption of introgression between modern and archaic forms of humans, which is proposed to have occurred only in the last 0.10 MY, provides a ready vehicle that would have transported archaic louse DNA into the modern louse population. Eswaran's model applied to lice suggests that at the beginning of the diffusion wave of modern H. sapiens leaving Africa (ca. 0.13 MYA), modern and archaic humans had distinct types of lice owing to ≥1 MY of isolation. As modern humans began to replace archaic forms, direct contact between hosts during introgression allowed archaic lice to switch to modern H. sapiens hosts.

As previously stated, the recent literature addressing human origins boils down to models that do not permit introgression (strict-sense replacement models) and those of many types that do (admixture models, including variants allied with both the Multiregional Evolution and Recent African Replacement models). All things being equal, our parasite data are most consistent with a limited amount of admixture between modern and archaic humans, because this process presents the opportunity for host switching. However, introgression between modern and archaic humans over a protracted period of time would erode the isolation required to maintain the two louse lineages that we have observed. For example, some variants of the Multiregional Evolution model reject a single origin of modernity in Africa ca. 0.13 MYA in favor of a piecemeal acquisition of modern traits over a long period of time. This long-term admixture is precisely what would disrupt the isolation required to maintain the two louse lineages. Eswaran's Diffusion Wave model, on the other hand, confines admixture to the last ca. 0.10 MY.

Our data cannot directly address whether host introgression occurred, because nonsexual, direct contact between hosts is sufficient for parasite transmission. We are confident that “direct contact” would be required for a host switch because these obligate parasitic lice cannot move between individuals without direct physical contact (Buxton 1946; Durden 2001; Canyon et al. 2002; Burgess 2004) and furthermore, they die within 24 h of being removed from their host. However, an examination of *Pthirus pubis,* the human pubic louse, might shed light on the subject of human admixture because unlike head and body lice, pubic lice are primarily transmitted during intercourse.

If our scenario involving lice switching from H. erectus to H. sapiens were true, then the host switch would have brought together two long-separated taxa of lice. It is impossible to know whether this long separation affected the reproductive compatibility of the two louse taxa once reunited. Discriminant function analysis shows no morphological differences between members of the two divergent molecular haplotypes of head lice (see Figure 3). There are other well-defined species of lice (e.g., see Johnson et al. 2002) whose populations show even greater sequence divergence (19% uncorrected sequence divergence) and yet have no discernible morphological differences between populations. It is likely that the two long-separated types of lice have experienced some level of introgression since their secondary contact on modern *H. sapiens.* The recency of this introgression of archaic louse DNA into modern lice also accounts for the younger coalescence time for the NW clade (0.15 MYA) compared to the WW clade (0.54 MYA). Presumably, the archaic form (i.e., morphotype) of louse either was extirpated along with its host or was assimilated into modern *P. humanus.* Regardless of the mechanism, ancient louse lineages can be found among the lice of modern *H. sapiens.*

A recent review by Ashford (2000) reported five parasites that occur on humans as closely related pairs of taxa (lice, tapeworms, follicle mites, a protozoan, and bedbugs). The fact that there are *five* such pairs caused Ashford to ask, were humans once two distinct populations that rejoined after a long separation? The ancient divergences seen in mitochondrial data from P. humanus are clearly consistent with some level of long-term host isolation, and preliminary evidence from nuclear markers (EF1-alpha and 18S rRNA) reveals similarly ancient divergences (unpublished data). Furthermore, the two tapeworm species from humans showed amazingly concordant divergences (Hoberg et al. 2001) and distributional patterns. Our data suggest that the isolation Ashford refers to may be between species of *Homo* rather than within modern H. sapiens itself. We conclude that the parasites may be very useful in the study of human evolutionary history, because they represent an independent marker of human evolution that has yet to be studied in detail.

## Materials and Methods

### 

#### Specimen collection and preparation.

We collected human head and body lice *(P. humanus)* from many localities, ranging from remote areas such as the Papua New Guinea highlands to metropolitan areas like Boston (Table 2). We also obtained P. schaeffi (from chimpanzees), Pthirus pubis (from humans), Pedicinus hamadryas (from baboons), and Fahrenholzia pinnata (from a rodent) to use as outgroup taxa in phylogenetic analyses. All lice were preserved in 95% EtOH and stored at −80 °C. DNA was extracted from lice by separating the thorax and abdomen and placing both in digestion buffer (Qiagen DNeasy tissue kit; Qiagen, Valencia, California, United States). Digestion proceeded for 48 h at 55 °C, then followed the manufacturer's protocol. After digestion, each louse was reassembled on a microscope slide as a voucher specimen corresponding to each DNA sequence. Voucher specimens were deposited in the Price Institute of Phthirapteran Research (PIPeR) collection at the University of Utah.

**Table 2:**
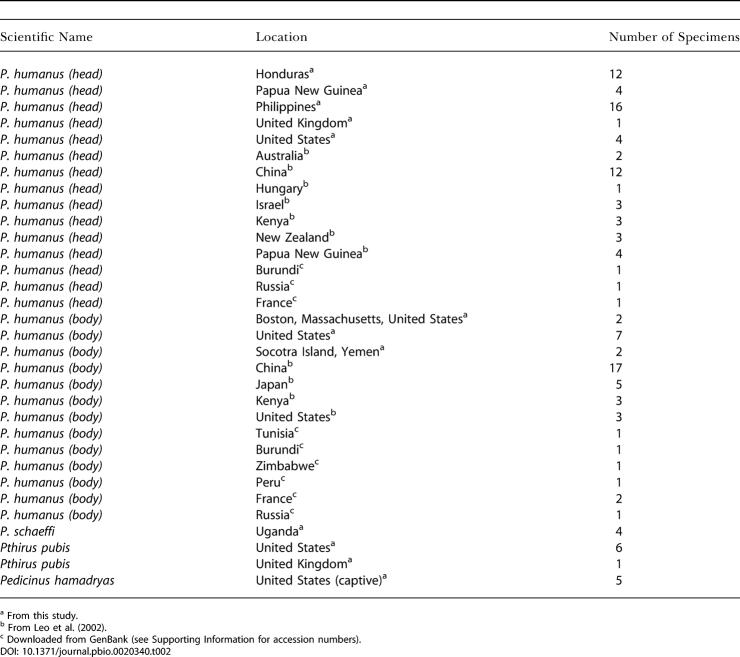
Specimens of P. humanus and Outgroup Taxa Examined in This Study, Their Collection Locality, and Number of Specimens Examined

^a^ From this study

^b^ From Leo et al. (2002)

^c^ Downloaded from GenBank (see Supporting Information for accession numbers)

#### Phylogenetic analyses: morphological data.

Considerable morphological variation exists among different species of primate lice. We examined 155 unordered morphological characters for 113 specimens of P. humanus (from humans), P. schaeffi (from chimpanzees), Pthirus pubis (from humans), Pthirus gorillae (from gorillas), Pedicinus hamadryas (from baboons), and F. pinnata (from a rodent). Morphological data were scored in the software package MacClade v. 4.05 (W. P. Maddison and D. R. Maddison; Sinauer, Sunderland, Massachusetts, United States), and heuristic searches consisting of random stepwise addition (1,000 replicates) and tree bisection/reconnection branch swapping were performed in PAUP* v. 4.0b10 (D. L. Swofford; Sinauer). Branch support was estimated with bootstrapping (tree bisection/reconnection swapping, 1,000 replicates). The complete data matrix is available from TreeBASE (http://www.treebase.org/) as study accession number SN1969.

#### Primate-louse cospeciation.

The morphological data set (six species, 155 characters) was compared to the host phylogeny ((((human, chimp), gorilla), baboon), rodent) using reconciliation analysis in Treemap v. 2.0 with default parameters. Treemap determines whether the two phylogenies are more congruent than expected by chance based on randomizations of both the host and parasite phylogeny. Significant congruence between host and parasite phylogenies is interpreted as being the result of a shared evolutionary history (i.e., repeated bouts of cospeciation).

#### Discriminant function analysis.

We examined the morphology of P. humanus lice in detail to test for morphological correlates of the differences detected at the molecular level. Busvine (1978) examined a large series of head and body louse specimens and found no discrete morphological differences between the two forms. However, he noted that several morphological characters related to size and shape might be useful in this regard. To test this hypothesis we measured head width, thoracic width, total body length, and second-leg tibia length from a series of 50 slide-mounted adult museum specimens collected by earlier workers prior to our study. The microhabitat (head or body, i.e., clothing) from which these museum specimens were collected was well documented. Canonical discriminant analysis was used to build a predictive model to attempt to distinguish between the head and body forms of *P. humanus.* The predictor variables were used to build a set of discriminant functions that maximized variation among groups while minimizing within-group variation. The first two canonical discriminant functions explained 100% of the variation within the data.

These discriminant functions, which were built using existing museum specimens, were then applied to our newly collected specimens in a blind test to determine whether the specimens could be identified as head or body lice from morphology alone. We were able to classify our samples as head or body lice with a probability of ≥0.95. Indeed, the assignment of adult specimens proved to be 100% accurate when checked against microhabitat data for the new specimens.

#### Phylogenetic analyses: molecular data.

Fresh specimens suitable for the collection of molecular data were obtained for five of the six species of lice. We sequenced 1,525 combined base pairs (bp) of the mitochondrial (mtDNA) genes COI (854 bp) and Cytb (671 bp) from 69 individuals of *P. humanus, P. schaeffi, Pthirus pubis, Pedicinus hamadryas,* and *F. pinnata.* PCR primers were as follows: (5′−3′) COI, C1-J-1718 GGAGGTTTTGCTAATTGATTAG and H7005 CCGGATCCACNACRTARTANGTRTCRTG; Cytb, L11122 GAAATTTTGGGTCWTTRCTNGG and H11823 GGCATATGCGAATARGAARTATCA. PCR parameters included 94 °C for 30 s, 48 °C for 30 s, and 72 °C for 1.5 min (five cycles), then 30 cycles of 94 °C for 30 s, 52 °C for 30 s, and 72 °C for 1.5 min. Amplified fragments were sequenced in both directions, assembled using Sequencher v. 4.1 (GeneCodes, Ann Arbor, Michigan, United States), and deposited in the NCBI database (see Supporting Information). To ensure that we were not amplifying nuclear copies of mitochondrial genes, we performed additional PCR amplifications using nested sets of overlapping primers.

The computer program modelTest (Posada and Crandall 1998) was used as a guide to determine a best-fit (Cunningham et al. 1998) ML model for the molecular data. This model (GTR+I+G) was incorporated into ML branch and bound and heuristic searches in PAUP* with 100 bootstrap replicates. An LRT was used to compare ML estimates from a clock-enforced and an unconstrained analysis. Our data did not depart significantly from the assumption of a molecular clock.

#### Dating nodes in the louse phylogeny.

We used the significant cospeciation shown between the primate and louse phylogenies (see Results) as a basis for dating nodes in the louse phylogeny. We used a calibration point of 22.5 ± 2.5 MYA for the split between *Pedicinus* and *Pediculus* + *Pthirus.* The date is based on fossil evidence (Benefit 1993; Leakey et al. 1995) of the split between cercopithecoid primates that host only lice in the genus *Pedicinus* and hominoid primates that host only lice in the genera *Pthirus* and *Pediculus* (Durden and Musser 1994). All reconciliations of the host and parasite trees in our cospeciation analysis determined that this particular host/parasite node represents a cospeciation event (see Results). Using the computer software r8s (M. J. Sanderson, software distributed by the author), we constrained the divergence of *Pedicinus* + *Pthirus/Pediculus* to 22.5 ± 2.5 MYA and allowed all other nodes in the louse tree to be determined from our DNA sequence data. Error estimates on divergence dates were calculated by generating 100 bootstrapped data matrices in Phylip (J. Felsenstein, software distributed by the author). Each of these bootstrapped datasets was calibrated with the same 22.5 ± 2.5 MYA divergence.

#### Cospeciation within *Homo*

An ML-based analysis was used to determine whether deep divergences in the louse tree were contemporaneous with divergences of now extinct species of *Homo.* We calculated the branch length between H. sapiens and H. neanderthalensis (the only species of *Homo* for which DNA sequence data are available) based on the human mitochondrial hypervariable region II of the D loop (see Supporting Information). This value was scaled according to the average distance between these two taxa and their sister taxon (chimpanzee), providing a relative branch length within the primate tree (e.g., the branch between human and Neandertal is one-fifth the length of the branch that unites them with the chimpanzee). This relative branch length was incorporated into a louse constraint tree, in effect forcing the two clades of P. humanus to conform to a prescribed relative branch length. The resulting likelihood score was compared with the unconstrained tree score using an LRT (d.f. = taxa − 2). If the constrained and unconstrained tree scores are not significantly different, then the host tree topology and branch lengths describe the parasite DNA sequence data as well as the parasite tree itself (Huelsenbeck and Crandall 1997). However, if a significant difference is detected, then the host tree does not fit the parasite data well enough to be explained by cospeciation, and the hypothesis of cospeciation is rejected.

#### Population genetic analyses.

Population genetic analyses were performed on a pruned dataset, which contained only specimens of P. humanus (i.e., no outgroup taxa). The computer software package DnaSP (Rozas and Rozas 1999) was used to generate mismatch distributions, to calculate Fu and Li's D* statistic for the P. humanus clades, and to calculate additional population parameters (e.g., F_st_ and Θ). Ten additional haplotypes from Leo et al. (2002) were used in these population-level analyses (see Supporting Information). To estimate an expected time to coalescence for *P. humanus,* we used estimates of theta (Θ, from the software package DnaSP [Rozas and Rozas 1999]) and mutation rate (μ) for P. humanus (see below) to calculate louse N_ef_ from the equation Θ = 2N_ef_μ. The mutation rate (μ = 9.0 × 10^−9^ substitutions per site per generation) was calculated by determining the expected number of substitutions per site between P. humanus and P. schaeffi under the Tamura-Nei + Γ model of nucleotide substitution. This mutation rate for P. humanus is roughly five to six times faster than that of human mtDNA, excluding the D-loop (Ingman et al. 2000), when both mutation rates are scaled to absolute time (i.e., number of substitutions per site per year). N_ef_ was then used to determine the expected time to coalescence (in generations) given the formula 2N_ef_ (1 − 1/*n*), where *n* is the number of haplotypes detected in the population. One can also ask the similar question, what is the probability that two lineages, which are expected to differ by k_e_ substitutions, actually differ by k^*^ or more substitutions? The expression used, (k_e_ /(1+ k_e_) ^ k^*^), is derived from the geometric distribution (see, for example, Golding and Strobeck 1982) and for our data suggests that the large number of substitutions found between the WW and NW clades is far greater than that which is expected (*p* < 0.0006).

## Supporting Information

### Accession Numbers

GenBank (http://www.ncbi.nlm.nih.gov/Genbank/) accession numbers for items discussed in the text are as follows: the EF1-alpha sequences from Kittler et al. (2003), AY316794–AY316834; the 18S rRNA sequences for P. humanus from Yong et al. (2003), AY236410–AY236418, AF139478–AF139482, AF139484, AF139486, and AF139488; the 18S rRNA sequence from *P. schaeffi,* AY695939; the Cytb sequence from *P. schaeffi,* AY316793; the human mitochondrial hypervariable region II of the D loop, M76311, AY195756, AY217615, AF282972, AF142095, X97709, X98472, X93336, X93337, X93347, and X93348; the ten haplotypes from Leo et al. (2002) used in population-level analyses, AF320286; the divergent louse Dennyus hirundinus shown in Figure 4, AF545694 and U96434.

The amplified PCR fragments discussed in Materials and Methods have been deposited in the NCBI database under accession numbers AY695939–AY696069.

## Abbreviations

COI: cytochrome oxidase subunit I
Cytb: cytochrome b
HTLV: human T-cell leukaemia/lymphoma virus
LRT: likelihood ratio test
ML: maximum likelihood
MRCA: most recent common ancestor
mtDNA: mitochondrial DNA
MY: million years
MYA: million years ago
N_ef_: effective female population size
NW: New World
WW: worldwide
